# Management of Microvascular Bleeding after On-Pump Cardiac Surgery in a Patient with Perioperative Diagnosis of Impairment of Platelet Responses to Adenosine Diphosphate: A Case Report and a Literature Review

**DOI:** 10.3390/jcm12196372

**Published:** 2023-10-05

**Authors:** Jacopo D’Andria Ursoleo, Margherita Licheri, Gaia Barucco, Sara Breggion, Francesco De Simone, Fabrizio Monaco

**Affiliations:** 1 Department of Anesthesia and Intensive Care, IRCCS San Raffaele Scientific Institute, Via Olgettina 60, 20132 Milan, Italy; dandria.jacopo@hsr.it (J.D.U.); licheri.margherita@hsr.it (M.L.); barucco.gaia@hsr.it (G.B.); desimone.francesco@hsr.it (F.D.S.); 2 San Raffaele Telethon Institute for Gene Therapy (SR-Tiget), IRCCS San Raffaele Scientific Institute, Via Olgettina 60, 20132 Milan, Italy; breggion.sara@hsr.it

**Keywords:** adenosine diphosphate, blood platelets, cardiac surgical procedures, cardiopulmonary bypass, deamino arginine vasopressin, P2Y_12_ purinoceptor

## Abstract

Background: Impairment of platelet responses to adenosine diphosphate (ADP) is typified by mild to severe bleeding diathesis, easy bruising, excessive mucosal and post-operative bleeding. Patients lack full platelet activation and aggregation in response to ADP. Following research of the literature in Scopus, PubMed/MEDLINE, ScienceDirect, and the Cochrane Library, we report only 18 patients described to date with impaired platelet response to ADP, none of whom in the high bleeding-risk surgical setting or exploring potential therapeutic options. Data regarding population, putative genetic mutations, modes of inheritance, functional defects, and related clinical manifestations were retrieved from case series and case reports. Case presentation: A 40-year-old woman was scheduled for on-pump cardiac surgery. Her past medical history included episodes of spontaneous mucocutaneous hemorrhages of the mild entity since childhood. Multiple electrode aggregometry (MEA, Multiplate^®^ Roche Diagnostics, Rotkreuz, Switzerland) was used to evaluate platelet response to thrombin-activated peptide-6 (TRAP), arachidonic acid (ASPI), and ADP. An inadequate platelet aggregation induced using a high concentration of ADP with normal TRAP and ASPI tests was detected preoperatively. Therefore, intravenous desmopressin (DVVAP) 0.3 μg/kg body weight was administered to manage microvascular bleeding developed after weaning from cardiopulmonary bypass (CPB). Conclusions: Proper management of impaired platelet response to ADP requires a systematic assessment. The Multiplate analyzer is a valuable tool to promptly detect the disorder when a high clinical suspect is present and obtain insights during high bleeding-risk surgical procedures. DVVAP can be beneficial as first-line therapy in bleeding patients to improve platelet function.

## 1. Introduction

Qualitative or quantitative defects of platelet P2Y_12_ receptor lead to impaired platelet responses to adenosine diphosphate (ADP), a rare condition characterized by a reduced ability of platelets to aggregate and form blood clots in response to ADP stimulation. This condition belongs to the more heterogeneous group of platelet function disorders (PFD), characterized by dysfunctional platelets exhibiting decreased function due to abnormalities either in the components engaged in signal transduction (e.g., membrane receptors, granules, etc.) or in their biochemical machinery [1].

The clinical presentation of impairment of platelet responses to ADP spans from easy bruising and mild to severe spontaneous bleeding diathesis (e.g., epistaxis and menorrhagia) to excessive post-operative or post-traumatic blood loss. In such cases where platelet function is impaired, the disease’s most typical feature is ADP failing to induce stable and full platelet aggregation, even at very high concentrations (≥10 μM). Moreover, dysfunctional platelets exhibit compromised inhibition of cyclic adenosine monophosphate (cAMP) increase, which further impairs platelet aggregation. Of note, platelet count and morphology are normal [2,3].

The actual incidence of PFD is unknown, but it is estimated that between 1:10,000 and 1:1,000,000 individuals are affected [1]. Substantial clinical and laboratory heterogeneity, together with limited reproducibility and specificity of platelet function tests, has historically impacted negatively on the agility of PFD diagnostic workup. Therefore, the disease prevalence is probably underestimated. 

Diagnosis generally benefits from a comprehensive clinical evaluation of the patient, followed by preliminary laboratory investigations and light transmission aggregometry (LTA) to evaluate platelet response to a limited or, if needed, expanded agonist spectrum. Cases with a strong suspicion of IPFD are then further investigated by molecular genetic studies that are available only at a few specialized centers and might require a notable quantity of blood samples [4].

Early identification of impaired platelet responses to ADP is crucial in patients scheduled to undergo high bleeding-risk surgical procedures. Nevertheless, major advances in the clinical management workup of PFD are still lacking. Current recommendations include educational and preventive measures to minimize the risk of bleeding (e.g., avoiding activities prone to trauma, maintaining good dental hygiene, and refraining from antiplatelet drugs). Hemostatic drugs, such as antifibrinolytic agents or desmopressin acetate (1-deamino-8-d-arginine vasopressin; DDAVP), are used to manage bleeding complications, based on both their severity or risk level [5,6]. 

The formers delay clot lysis by saturating the fibrin-binding sites on plasminogen, ultimately stabilizing clot formation, while the latter seems to enhance the ability to form procoagulant platelets and, by promoting Na^+^/Ca^2+^ mobilization, to implement platelet-dependent thrombin generation. 

Remarkably, no cases of impaired platelet responses to ADP have been reported so far in the high bleeding-risk setting of cardiac surgery.

In this investigation, we show that the diagnosis of impairment of platelet responses to ADP can be performed preoperatively via multiple electrode aggregometry (MEA) and that DDAVP may be effective as first-line therapy to treat microvascular bleeding in the on-pump cardiac surgery setting. 

We also present a summary of other cases and a review of the literature regarding the diagnosis and treatment of impaired platelet responses to ADP.

## 2. Materials and Methods

### 2.1. Study Design and Search Strategy

We performed a literature search in the electronic databases Scopus, PubMed/MEDLINE, and ScienceDirect from their inception to June 2023 to carry out a review of the literature. Keywords “P2Y_12_” and “defect” were combined using the Boolean operator “AND”.

### 2.2. Inclusion Criteria

The PICO (P: patient/population/problem, I: intervention, C: comparison/control—O: outcome) framework was used to formulate our research question. Population: patients with intrinsic inability of ADP to elicit complete and irreversible platelet aggregation even at extremely high concentrations (>10 μM); intervention: pharmacological treatment for patients with impaired platelet response to ADP undergoing surgery at high risk of perioperative bleeding; comparators: no comparators; outcomes: summarizing the available evidence to date regarding cases of impairment of platelet responses to ADP and to identify the most frequently diagnostical and therapeutic approaches. Studies reporting original data (e.g., case reports and case series) in a pertinent setting were included. Original investigations in a non-pertinent setting (e.g., in vitro and cellular models, etc.), investigations that reported non-original data, and literature published in any language other than English were excluded.

### 2.3. Study Selection and Data Extraction

Titles and abstracts from the studies in the search result were independently screened by one author (JDAU) who extracted the following data as well: study features (authors, year of publication), population (number of cases), genetic characteristics of the disease (putative mutation, location and mode of inheritance) with the related clinical characteristics of the disease (degree of bleeding tendency, residual platelet function), diagnostical features and type of treatment (if specified). Data extraction was double-checked by another author (FM), who also provided valuable clinical and scientific insights. 

### 2.4. Data Synthesis

Data from the included studies were extracted using a standardized form. The following features were evaluated: genetic abnormality, location and mode of inheritance, resulting platelet functional defect, and related clinical manifestations.

## 3. Results

### 3.1. Case Presentation

A 40-year-old woman with severe aortic valve regurgitation, increased fatigue, and dyspnea (NYHA IIb) was scheduled for open minimally invasive aortic valve replacement. Her medical history was significant for hypertension, epileptic seizures, and spontaneous mucocutaneous hemorrhages of the moderate entity since childhood, with no familial background of hematological disorders. Preoperative standard laboratory tests were normal and revealed a white blood cell count of 6.7 × 10^9^/L, red blood cells 4.37 × 10^12^/L, hemoglobin 12.1 g/dL, hematocrit of 43.7%, and platelets (PLT) 210 × 10^9^/L. Basic hemostasis tests, including prothrombin time (PT), International Normalized Ratio (INR), partial thromboplastin time (PTT)-LA screen, PT-LA mixing studies, and antithrombin III (ATIII) activity levels, were in range as well.

**Figure 1:**
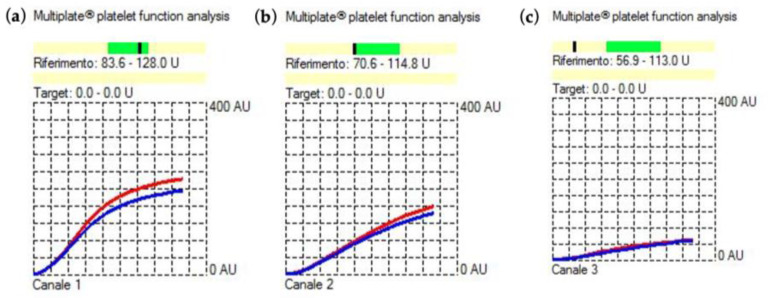
Results of the baseline preoperative functional platelet assay with (**a**) thrombin-activated peptide-6 (TRAP), (**b**) arachidonic acid (ASPI), and (**c**) adenosine diphosphate (ADP) tests using multiple electrode aggregometry (MEA, Multiplate^®^ Roche Diagnostics). The normal reference interval for the TRAP, ASPI, and ADP tests is represented by the darker green field above the X-axis. If in the darker fields, the black dash indicates normal platelet function. On the contrary, if the black dash falls beneath, it indicates impaired platelet function. Blue and red curves result from the test sample being analyzed in each test cell using two independent sensors. The difference should not exceed 15%. All tests were within this variation.

Preoperative platelet function was further tested with a Multiplate^®^ (Roche Diagnostics, Mannheim, Germany) analyzer in light of the patient’s reported long history of bleeding diathesis in the absence of pathological results for both standard laboratory and coagulation tests. It revealed normal platelet response to thrombin-activated peptide-6 (TRAP) and arachidonic acid (ASPI) (Figure 1a,b) and reduced platelet aggregation induced using high concentration (20 μL) of ADP. The ADP showed an area under the curve (AUC) of 23 U (Figure 1c). 

The cardiac surgery procedure was managed as per routine hospital protocol, which encompassed a ROTEM-enhanced transfusion protocol proved to reduce the need for intraoperative transfusion of allogenic blood products and administration of lower doses of protamine to minimize potential paradox bleeding from protamine overdose [7,8].

Prior to the establishment of cardiopulmonary bypass (CPB), the patient was administered 3.3 mg/Kg of unfractionated heparin (UFH), subsequently reverted after CPB discontinuation with 150 mg of protamine as per hemostasis management system (HMS Plus).

**Figure 2:**
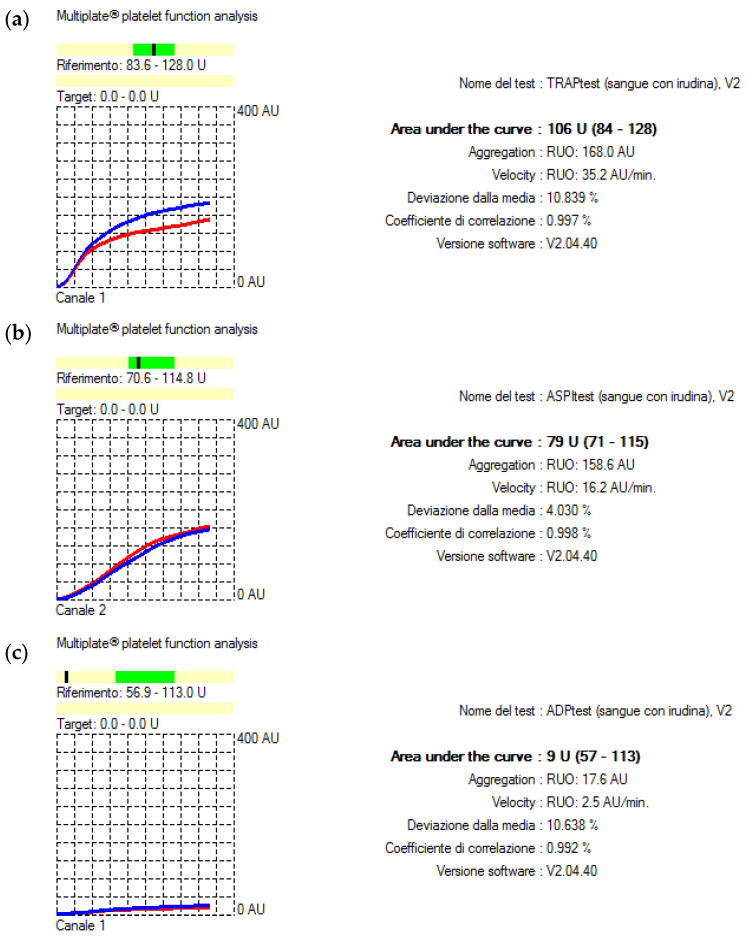
Results of the intraoperative functional platelet assay with (**a**) TRAP, (**b**) ASPI, and (**c**) ADP tests following weaning from CPB using MEA (Multiplate^®^ Roche Diagnostics). The normal reference interval for the TRAP, ASPI, and ADP tests is represented by the darker green field above the X-axis. If in the darker fields, the black dash indicates normal platelet function. On the contrary, if the black dash falls beneath, it indicates impaired platelet function. Blue and red curves result from the test sample being analyzed in each test cell using two independent sensors. The difference should not exceed 15%. All tests were within this variation.

After protamine administration, Multiplate analysis showed an overall worsened platelet function (Figure 2a–c). 

For the management of microvascular bleeding subsequent to separation from CPB and administration of protamine, an infusion of desmopressin at a dose of 0.3 μg/kg body weight in 50 cc saline was administered over a 30-minute period. Platelet transfusion was held in reserve solely as a therapeutic option in the event of refractory bleeding [5]. 

**Figure 3:**
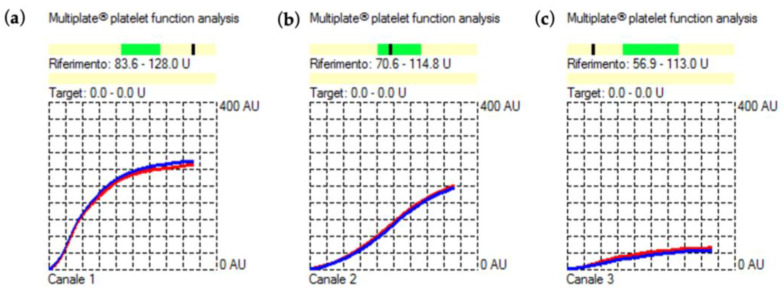
Results of the intraoperative functional platelet assay with (**a**) TRAP, (**b**) ASPI, and (**c**) ADP tests following intravenous infusion of desmopressin 0.3 μg/kg body weight using MEA (Multiplate^®^ Roche Diagnostics). The normal reference interval for the TRAP, ASPI, and ADP tests is represented by the darker green field above the X-axis. If in the darker fields, the black dash indicates normal platelet function. On the contrary, if the black dash falls beneath, it indicates impaired platelet function. Blue and red curves result from the test sample being analyzed in each test cell using two independent sensors. The difference should not exceed 15%. All tests were within this variation.

The Multiplate analysis performed after desmopressin administration showed a significant improvement in the TRAP, ASPI, and ADP tests AUC that were 166U, 84U, and 27U, respectively (Figure 3a–c).

Once a satisfactory hemostasis was achieved, the sternum was closed, and the surgical procedure concluded without further complications. The patient was extubated after 6 h in the intensive care unit (ICU). Overall drainage output was 400 mL without the need for transfusions. Additionally, no adverse effects attributable to the known desmopressin antidiuretic and vasomotor activity were recorded. She was discharged from the ICU on post-operative day 3 and from the hospital on day 8.

### 3.2. Review of the Literature

**Figure 4:**
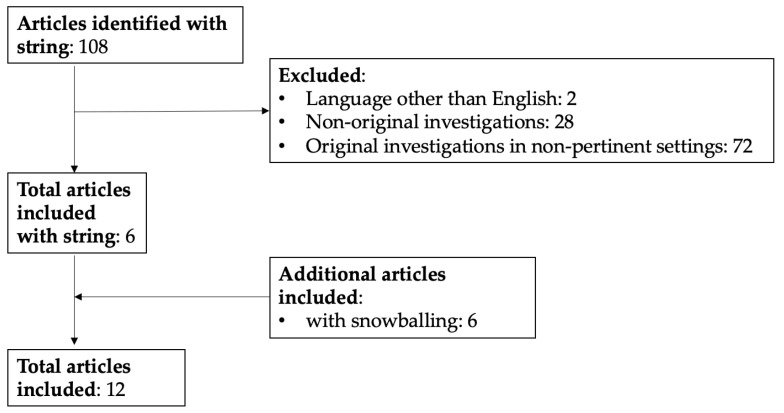
Flow diagram of studies’ selection.

The search strategy retrieved 108 items. After the removal of articles not suitable for inclusion (*n* = 102) and the addition of more articles identified by means of backward and forward snowballing (*n* = 6), a total of 12 articles were retained for data extraction. The flowchart of study selection is shown in Figure 4.

#### Historical Findings

The first online available case report of impairment of platelet responses to ADP dates back to 1992, when Cattaneo described a man with bleeding tendency and easy bruising due to the inability of ADP, even at extremely high concentrations (>10 μM), to elicit complete and irreversible platelet aggregation, transmitted as an autosomal recessive trait [9]. 

Nurden then studied a patient with a family history of bleeding and reported the same selective aggregation impairment, together with failed inhibition of cyclic adenosine monophosphate (cAMP) levels induced using prostaglandin E_1_ (PGE_1_) [10]. 

Cattaneo further described two sisters with identical ADP receptor abnormality and moderate to severe bleeding complications after dental extractions or major surgery [11]. Fourteen more cases were documented from 2003 to 2022, and several P2Y_12_ variants were identified using locus analysis. Interestingly, such P2Y_12_ defects can be qualitative or quantitative (Table 1).

The genetic study of one of the two aforementioned sisters’ sons led to the characterization of P2Y_12_ gene haploinsufficiency, where a single allele encodes a normal P2Y_12_. The partial defect yielded abnormal aggregation and adenosine triphosphate (ATP) secretion induced using several agonists, moderate deficiency of platelet-binding sites for an ADP analog, and partial impairment of inhibition of adenylate cyclase by ADP [16]. 

Heterozygous single base-pair substitutions were identified in five more patients. Cattaneo described a Japanese woman compound heterozygous for Arg256Gln in transmembrane domain 6 (TM6) and for Arg265Trp in extracellular loop 3 (EL3) of P2Y_12_. The structural integrity of these regions is necessary for normal receptor function since TM6 may represent a switch for the activation state of the receptor [21], and EL3 amino acids are important for receptor function or subtype specificity [22]. Both variants did not interfere with receptor surface expression but depleted its activity upon ADP stimulation, with lower and rapidly reversible platelet aggregation, impaired ATP secretion, and ADP effect on inhibition on PGE_1_-induced increase in cAMP levels [12]. 

Remijn and Daly reported two further mutations falling in extracellular loops, Pro258Thr in EL3 and K174E in EL2, respectively, confirming that these regions are important for receptor function [14,15]. 

Daly reviewed 92 patients enrolled in both the European Molecular and Clinical Markers for the Diagnosis and Management of Type 1 von Willebrand Disease (MCMDM-1VWD) study and the Canadian Type 1 VWD Study to dissect whether abnormalities in the P2Y_12_ ADP receptor gene contributed to the probands’ bleeding tendencies [23]. They discovered one index case which, together with their two relatives, carried the same mutation affecting the platelet P2Y_12_ receptor [15]. 

In 2011, Nisar investigated the functional role of the C-terminus of P2Y_12_ receptor [17]. Similarly to Daly, she screened the same cohort of patients [15] and identified one case with a predictive substitution falling in the post-synaptic density 95/disc large/zonula occludens (PDZ)-binding motif. The patient was unavailable to undergo further analysis, but the mother was found to carry the same missense mutation within the above-mentioned (PDZ)-binding motif affecting P2Y_12_ receptor recycling but not impacting aggregation and dense granule secretion [17]. 

The last heterozygous substitution identified so far is a D > N switch within the P2Y_12_ DRY motif, which impairs platelet aggregation in response to an ADP analog [20]. In 2004, Patel was the first to report a substitution, describing a R122C homozygous mutation in the DRY motif, which affected receptor signaling, traffic, and surface expression [18].

Shiraga first described a case of congenital P2Y_12_ deficiency owing to a homozygous mutation in the translation initiation codon in the receptor gene [13]. 

Lecchi reported a further mutated P2Y_12_ transmembrane domain in two brothers harboring p.His187Gln substitution in the TM5 portion of P2Y_12_, which plays a significant role in agonist and nucleotide antagonist binding. She identified the same mutation in a further six subjects within the same family, though two sisters only presented with a positive history of mild bleeding. Nonetheless, the probands solely contributed blood samples for genetic assessment, lacking the opportunity to conduct platelet function analysis. [19]. 

Interestingly, none of the aforementioned authors provided information on the defect’s therapeutic management, even if some described patients’ bleeding tendency upon surgical challenge [10,11,12,18,19].

## 4. Discussion

In the present case report, we introduce several novel findings. Firstly, platelet function assays should be conducted when available as part of the perioperative practice, even for patients with mild history of spontaneous hemorrhages and no prior diagnosis of a bleeding disorder before they undergo surgery at high risk of perioperative bleeding. Secondly, MEA is sensitive in identifying impairment of platelet responses to ADP. Thirdly, once the diagnosis of a platelet functional disorder is confirmed, it is both logical and potentially more advantageous to administer a pharmacological targeted therapy and avoid transfusions. We also present evidence, for the first time in the cardiac surgery setting, that intraoperative desmopressin infusion is an effective treatment for microvascular bleeding in patients with impaired platelet response to ADP.

Human platelets show two synergistic receptors for ADP: P2Y_1_, a G_q_-coupled receptor, which triggers platelet shape changes and ADP-induced aggregation upon the mobilization of internal Ca^2+^ stores, and P2Y_12_, a G_i_-coupled receptor involved in adenylyl cyclase and phosphoinositide 3-kinase activation (PI3K) [2]. Normal aggregation requires the activation of both receptors, yet ADP engagement with the P2Y_12_ receptor amplifies platelet response, preserves platelet aggregates, and contributes to the procoagulant function of platelets [3]. 

Aside from platelets, the P2Y_12_ receptor is expressed in many other cell types, including endothelial, glial, and smooth muscle cells [2]. The receptor presents agonists and antagonists, which can be of aid when investigating its potential molecular defects. ADP and analogs (e.g., 2-methylthio-ADP (2-MeS-ADP) or (N)-methanocarba-2MeSADP (MRS2365)) act as its potent agonists. On the contrary, ATP and derivatives (e.g., AR-C69931X and AZD6140) are classified as antagonists [24].

Due to its low prevalence, though, the diagnosis of impaired platelet responses to ADP is typically made via exclusion. The preliminary diagnostic phase involves a comprehensive clinical assessment of the patient. This evaluation encompasses personal and familial bleeding history, with a specific focus on easy bruising, post-invasive procedures and dental extraction, blood loss, epistaxis, and menorrhagia. Additionally, the medical history should examine the potential impact of drug or food intake on platelet function. An exhaustive physical examination is required to detect any typical manifestation (e.g., spontaneous bleeding manifestations) of the disorder, and the patient should undergo laboratory investigations if the initial assessment reveals evident abnormalities. Consequently, in cases where both the full blood count and standard coagulation (e.g., PT, activated partial thromboplastin time (aPTT) and von Willebrand factor (vWF)) screening tests yield normal results, there should be a high suspicion of a PFD. To investigate this hypothesis further, it is advisable to perform platelet function assays (e.g., by means of light transmission aggregometry (LTA) using a limited set of agonists initially) if available on-site. If needed, an expanded panel of agonists can be employed for a more comprehensive assessment [3,4]. 

Currently, there are various methods that are useful to assess platelet function. Although LTA is considered the “gold standard” diagnostic test for evaluating platelet response to agonists in vitro, its practical application in daily clinical practice exhibits some drawbacks and is limited due to sample requirements, its time-consuming nature, and poor standardization. 

Other possibilities include performing the platelet function analyzer (PFA)-200, which simulates primary hemostasis and is endowed with high specificity for P2Y_12_ signaling using means of stimulation of adenylyl cyclase. Even if it allows monitoring of moderate to severe P2Y_12_ impairment, its application is mainly limited to the severe forms of such congenital defects since the test proved to be insensitive to mild ones [25]. 

A more attractive alternative to be employed in the clinical setting is MEA using the Multiplate^®^ analyzer to measure platelet function in whole-blood samples. This major convenience allows fast analysis and preserves the cellular environment since no centrifugation step is required. Beyond assessment of platelet function, it offers advantages in everyday clinical practice, such as the possibility to supervise patients treated with antiplatelet therapies and recognize bleeding diathesis. In the surgical context, it also allows for the perioperative assessment of bleeding risk, both before and during the surgical procedure [26]. Sibbing et al. compared MEA and LTA platelet aggregation analysis. They selected a cohort of patients scheduled for coronary angiography and analyzed whole blood samples before and after Clopidogrel administration. Interestingly, they reported a strong correlation between ADP-induced platelet aggregation measurements obtained with MEA on the Multiplate^®^ analyzer and the gold standard LTA [27]. This supports the use of the former method to early identify patients at high risk of bleeding and enable a tailored and effective therapeutic strategy. However, the results of the two methods proved to be comparable yet not fully identical since the techniques mechanistically differ from each other. Therefore, further investigations are needed to better characterize this technique’s potential to early diagnose PFD compared to LTA [28].

Management of impairment of platelet responses to ADP in the perioperative setting is challenging. While conservative and local hemostatic measures (e.g., pressure, topical thrombin/fibrin sealant and/or antifibrinolytic, etc.) can be effectively employed when minor bleeding (e.g., epistaxis) occurs, more potent and systemic homeostatic agents are available when the former is not effective, including DDAVP, platelet transfusions and recombinant human activated factor VII (rFVIIa). No clear management guidelines or consensus statement is available regarding severe bleeding management in patients affected by PFD in the intra- and post-operative period. Furthermore, data from the cardiac surgery setting is still lacking. Platelet transfusion represents the established therapeutic approach for surgical prophylaxis and management of moderate to severe bleeding in patients with qualitative and non-immune quantitative platelet defects. However, administering platelet concentrates carries potential risks such as immunomodulation, allergic reactions, and transmission of blood-borne pathogens [5]. 

Desmopressin, a synthetic analog of vasopressin often effective in mild-moderate bleeding in PFD, can be appealing even in this setting. When administered intravenously, it achieves its peak effect in 30–60 min and rapidly exhibits pleiotropic effects that enhance ex vivo platelet aggregation. These include increasing the concentration of circulating high molecular weight von Willebrand factor (vWF) multimers, as well as mobilizing Na^+^/Ca^2+^ within platelets, thereby improving their ability to form procoagulant structures. Additionally, desmopressin enhances platelet-vascular subendothelium interaction, triggers the expression of activation-dependent platelet antigens, and induces the formation of platelet microparticles [5]. Interestingly, among several prophylactic treatments studied in patients with PFD, desmopressin emerged as the most effective option alone or in combination with antifibrinolytic agents. It showed the lowest bleeding rates, while antifibrinolytic agents alone were significantly less effective [29]. We report that the intravenous administration of 0.3 μg/kg body weight of DDAVP [5] is effective in the management of patients with impairment of platelet responses to ADP undergoing high bleeding-risk surgery.

As demonstrated in this case report, DDAVP improved the platelet response to TRAP, ASPI, and ADP, as assessed using measurements obtained using MEA. The present investigation once again demonstrated that blood-free cardiac surgery is possible even in the event of microvascular bleeding in a patient with impaired platelet response to ADP.

Noteworthy, in the present case report, intravenous administration of desmopressin facilitated a successful and uneventful course of a minimally invasive aortic valve replacement procedure in a patient with innate bleeding diathesis who, at the same time, would not have benefited from transcatheter aortic valve replacement (TAVR) being aged <65 years and with life expectancy largely exceeding >20 years, as per the ACC/AHA recommendations [30].

## 5. Conclusions

Impaired platelet response to ADP should be in the differential diagnosis for patients with a history of mucocutaneous bleeding and normal laboratory tests once more common conditions are ruled out. 

MEA analysis using the Multiplate analyzer can effectively detect PFD and provide valuable preoperative information for high bleeding-risk surgical procedures. 

DVVAP offers a viable and promising therapeutic option in the event of microvascular bleeding during high bleeding-risk surgery, as it enhances platelet function and promotes hemostasis.

## Figures and Tables

**Table 1:** Summary of the main genetic mutations, modes of inheritance, functional defects, and related clinical manifestations in 18 patients with impaired platelet response to adenosine diphosphate (ADP) reported in the literature to date (1992–2022).

1st Author, Year	Genetic Abnormality	Domain	Mode of Inheritance	Functional Defect	Clinical Manifestations
Cattaneo et al., 1992 [9]	p.Gln98fs	Not identified	Likely autosomal recessive	Defective aggregation induced using ADP, epinephrine, PAF-acether, U46619, collagen, and arachidonic acid Impaired release of ^14^C-serotonin Partial inhibition of aggregation by ADP scavengers Defective fibrinogen and vWF binding induced using exogenous ADP ADP does not accelerate Anti-GPIIb/IIIa mAbs binding to platelets ADP prevented PGE_1_-induced increase in platelet cAMP	Mucosal bleeding Easy bruising Need for transfusion after accidents Nonsteroidal anti-inflammatory drugs worsened the bleeding diathesis
Nurden et al., 1995 [10]	p.Phe240fs. No mutation was identified in one of the two alleles	Not identified	Not specified	Limited and always reversible ADP-mediated platelet aggregation Failure in PGE_1_-induced cAMP decrease in response to ADP Markedly reduced platelet binding of [^3^H]2-MeS-ADP Impaired binding of fibrinogen to ADP-stimulated platelets Aggregates comprising a small number of loosely bound platelets with few contact points Weak response to Anti-GPIIb/IIIa mAbs	Brief episodes of excessive bleeding Excessive post-surgical bleeding
Cattaneo et al., 2000 [11]	378delC; p.Thr126fs	TM3	Likely autosomal recessive	Small and reversible wave of platelet aggregation in PRP induced using ADP or PAF-acether Impaired platelet secretion induced using agonists Failure in PGE_1_-induced cAMP decrease in response to ADP Severely decreased number of binding sites in platelets for [^33^P]2 MeS-ADP	Easy bruising Menorrhagia Severe bleeding complications after dental extractions or major surgery
					Easy bruising Epistaxis Menorrhagia Moderate bleeding complications after dental extractions
Cattaneo et al., 2003 [12]	p.Arg256Gln; p.Arg265Trp	TM6 and EL3	Compound heterozygous	Markedly lower and rapidly reversible ADP-induced platelet aggregation P2Y_12_ antagonist ARC69931MX is ineffective on platelets Weak or absent ATP secretion from platelets stimulated with ADP and other agonists Failure in PGE_1_-induced cAMP decrease in response to ADP	Easy bruising Excessive post-traumatic and post-surgical blood loss
Shiraga et al., 2005 [13]	A*T*G > A*G*G	translation initiation codon	Homozygous	Transient platelet aggregation induced using ADP, collagen, U46619, or PAR1 TRAP P2Y_12_ antagonist ARC69931MX is ineffective on platelets Abnormal inhibitory effect of ADP on PGE_1_-stimulated cAMP accumulation in platelets Impaired spreading on immobilized fibrinogen Formation of unstable thrombi on type 1 collagen	Easy bruising Mild bleeding tendency Massive bleeding during delivery
Remijn et al., 2007 [14]	p.Pro258Thr	EL3	Heterozygous	Impaired or absent platelet aggregation induced using ADP and collagen Small thrombi consisting of a spread and incomplete layer of platelets on top after perfusion of blood	Severe epistaxis Easy bruising Excessive post-traumatic blood loss
Daly et al., 2009 [15]	p.Lys174Glu	EL2	Heterozygous	Reduced and transient platelet aggregation to ADP Reduction in the maximal level of dense granule secretion induced using ADP and PAR-4 peptide in platelets Reduction in ADP and PAR-4-induced ATP secretion Reduced P2Y_12_ binding to [^3^H]2-MeS-ADP	Mild bleeding
Fontana et al., 2009 [16]	Haploinsufficiency of P2Y_12_ gene (single allele encodes a normal P2Y_12_)	Not identified	Not specified	Abnormal aggregation and ATP secretion induced using ADP, U46619, PAF-acether, or collagen Moderate deficiency of platelet-binding sites for [^33^P]2MeS-ADP Partial impairment of inhibition of adenylate cyclase by ADP	Mildly prolonged bleeding time
Nisar et al., 2011 [17]	p.Pro341Ala	PDZ-binding motif	Heterozygous	Pronounced reduction in P2Y_12_ surface binding sites in platelets Impaired inhibition of PGE_2_-stimulated adenylyl cyclase Sustained aggregation and dense granule secretion P2Y_12_ antagonist ARC69931MX is effective on platelets Impaired correct receptor recycling back to the membrane in human platelets	Mild bleeding tendency
Patel et al., 2014 [18]	p.Arg122Cys	DRY motif	Homozygous	Reduced platelet aggregation in response to ADP, PAR-1 peptide, or collagen Abrogated inhibition of pVASP phosphorylation Reduced cell surface P2Y_12_ expression T allele-associated decreased expression of PAR-1 on platelets, lower secretion responses to the PAR-1-activating peptide, and decreased procoagulant activity	Spontaneous bleeding Post-surgery hemorrhage
Lecchi et al., 2014 [19]	p.His187Gln	TM5	Homozygous	Normal expression of the receptor but decreased affinity for its ligand Completely suppressed function of the mutant receptor Straight and tilted TM5 Decreased affinity for [^3^H]PSB-0413 antagonist Normal number of platelet binding sites Reduced affinity for the P2Y_12_ receptor antagonist radioligand [^3^H]PSB-0413, ADP and 2MeSADP	Epistaxis Post-operative bleeding after tooth extraction
					Severe bleeding history
Dangelmaier et al., 2022 [20]	p.Asp121Asn	DRY motif	Heterozygous	Strongly inhibited response to 2-MeS-ADP Mild Akt phosphorylation at Ser473 Defects in both aggregation and secretion at low doses of AYPGKF Defective VASP phosphorylation and GTP-Rap1b formation Inhibited signaling downstream of Gi-coupled P2Y_12_ Reduced thrombus formation in whole blood	No bleeding diathesis

Gln: glutamine; fs: frameshift; ADP: adenosine diphosphate; PAF: platelet-activating factor; vWF: von Willebrand factor; GP: glycoprotein; mAbs: monoclonal antibodies; PGE_1_: prostaglandin E_1_; cAMP: cyclic adenosine monophosphate; Phe: phenylalanine; [^3^H]2-MeS-ADP: [^3^H]2-methylthio-adenosine diphosphate; del: deletion; Thr: threonine; TM: transmembrane domain; PRP: platelet-rich plasma; Arg: arginine; Trp: tryptophan; EL: extracellular loop; ATP: adenosine triphosphate; PAR: protease-activated receptor; TRAP: thrombin receptor-activating peptide; Pro: proline; Lys: lysine; Glu: glutamate; Ala: alanine; PDZ: post-synaptic density 95/disc large/zonula occludens-1; PGE_2_: prostaglandin E_2_; Cys: cysteine; pVASP: platelet vasodilator-stimulated phosphoprotein; His: histidine; [^3^H]PSB-0413: [^3^H]2-Propylthioadenosine-59-adenylic acid (1,1-chloro-1-phosphonomethyl-1-phosphonyl) anhydride; Asp: aspartate; Asn: asparagine; Akt: protein kinase B; Ser: serine; AYPGKF: PAR-4 agonist peptide.

## Data Availability

Further information is available from the corresponding authors upon reasonable request.
